# Risk factors for lower extremity deep vein thrombosis in acute stroke patients following endovascular thrombectomy: a retrospective cohort study

**DOI:** 10.3389/fneur.2023.1249365

**Published:** 2023-10-11

**Authors:** Li Han, Jian-Miao Yang, Wei-Yang Qian, Xiao-Ping Xu, Tao-Hsin Tung, Yang Liu, Feng Wang

**Affiliations:** ^1^Department of Neurology, Taizhou Hospital of Zhejiang Province, Affiliated to Wenzhou Medical University, Linhai, Zhejiang, China; ^2^Department of Pharmacy, Taizhou Hospital of Zhejiang Province, Affiliated to Wenzhou Medical University, Linhai, Zhejiang, China; ^3^Department of Neurosurgery, Taizhou Hospital of Zhejiang Province, Affiliated to Wenzhou Medical University, Linhai, Zhejiang, China; ^4^Evidence-Based Medicine Center, Taizhou Hospital of Zhejiang Province, Affiliated to Wenzhou Medical University, Linhai, Zhejiang, China; ^5^Department of Neurology, Saarland University, Homburg, Germany

**Keywords:** acute ischemic stroke, endovascular thrombectomy, deep vein thrombosis, risk factors, D-dimer (DD)

## Abstract

**Background:**

Deep vein thrombosis (DVT) in lower extremities as a common complication of acute ischemic stroke (AIS) has long been studied. However, as the therapeutic options for AIS continue to advance, the pathogenic mechanisms behind DVT may change. Endovascular thrombectomy (EVT) has replaced intravenous thrombolysis and become the preferred treatment for AIS patients with large vessel occlusions. Therefore, it is important to update our understanding of DVT and its management. This study aimed to determine the prevalence and risk factors of DVT in AIS patients following EVT.

**Methods:**

In this retrospective study, 245 AIS patients who had received EVT were recruited between January 2020 and December 2021. Within 10 days (median 4 days) of thrombectomy, DVT was diagnosed by ultrasonography. Demographic characteristics, clinical findings, and therapeutic procedures were compared between patients with and without DVT using univariate analysis. Cutoff points were defined for EVT time and plasma D-dimer concentration. Multivariable logistic regression was then used to determine the independent risk factors for DVT and evaluate their predictive power.

**Results:**

The prevalence of DVT in AIS patients after EVT was 27.3%. Multifactorial logistic regression analysis showed that age (OR 1.036, 95% CI 1.001–1.073; *P* = 0.045), female sex (OR 3.015, 95% CI 1.446–6.289; *P* = 0.003), lower limb muscle strength less than grade three (OR 7.015, 95% CI 1.887–26.080; *P* = 0.004), longer EVT time (OR 1.012, 95% CI 1.004–1.020; *P* = 0.003), and higher D-dimer levels (OR 1.350, 95% CI 1.150–1.585; *P* < 0.001) were independently associated with higher DVT risk in AIS patients following EVT. The cutoff points for operative time of EVT and plasma D-dimer were 65.5 min and 1.62 mg/L, respectively, above which the risk for DVT was dramatically increased with OR > 4 in AIS patients.

**Conclusion:**

AIS patients are at increased risk of developing DVT following EVT particularly if they have undergone prolonged thrombectomy procedures and exhibit high plasma levels of D-dimers. However, the results of our study need to be validated by a multicenter prospective study with a larger population of stroke patients.

## Introduction

Acute ischemic stroke (AIS) is one of the leading causes of disability and death both in China and worldwide, placing a heavy burden on both the healthcare system and families (1–3). One common complication in AIS patients is deep vein thrombosis (DVT), which is often diagnosed and monitored in the lower limbs. It was reported more than 40 years ago that up to 75% of stroke patients with hemiplegia without venous thromboembolism prophylaxis develop DVT in the lower limbs as evidenced by the uptake of ^125^I-labeled fibrinogen (4). Recently, duplex ultrasonography has become the standard method for diagnosing DVT, and the detected prevalence of DVT in low extremities ranged from 11.5 to 22.1% in stroke patients (5–10). DVT can lead to pulmonary embolism, which significantly damages the lungs and heart and is responsible for 13–25% of early deaths after stroke (11). DVT also leads to reduced mobility and prolonged hospitalization, which increases the risk of infection, pressure ulcers, and pneumonia. In addition, DVT can result in post-thrombotic syndrome, which causes chronic pain, swelling, and skin changes in the affected leg. This further impairs the patient's mobility and quality of life. Therefore, timely and effective diagnosis and treatment of DVT are necessary.

Occlusion of large proximal arteries supplying the brain leads to particularly poor functional outcomes in AIS patients (12). In 2015, several studies provided compelling evidence that endovascular thrombectomy (EVT) for stroke due to large vessel occlusion results in significantly better recanalization and clinical outcomes than intravenous thrombolysis alone (12). In 2019, the American Heart Association/American Stroke Association recommended EVT for AIS patients for up to 24 h after the onset of symptoms when imaging mismatch or mismatch between severity of clinical deficit and infarct volume is observed (13). EVT has now replaced intravenous thrombolysis and become a standard therapy for AIS patients with large vessel occlusions.

It should be noted that patients receiving EVT require general or local anesthesia during EVT and post-operative monitoring in the intensive care unit (ICU). The endovascular intervention may damage endothelial cells and induce thrombosis (14). The pathogenic mechanisms mediating DVT after EVT must be altered compared to those in previously studied stroke patients treated with and without intravenous thrombolysis. It is supposed that the prevalence of DVT in EVT-treated patients is higher. However, the exact prevalence/incidence and risk factors for DVT in EVT patients remain unclear. In this study, we aimed to answer this question by examining all stroke patients who received EVT in our hospital within 2 years.

## Methods

### Study design

Our project was a retrospective cohort study. The study protocol was approved by the Ethics Committee of Taizhou Hospital, Zhejiang Province, China (Registration number: K20181204). Between January 2020 and December 2021, all 310 AIS patients aged ≥18 years at our hospital received EVT according to the international and Chinese guidelines (13, 15).

The inclusion criteria were as follows: age ≥ 18 years; AIS diagnosis confirmed by computed tomography (CT) or magnetic resonance imaging (MRI); and EVT performed within 24 h of symptom onset. The exclusion criteria were as follows: no ultrasound examination, hospitalization of <3 days, death during hospitalization, no recanalization after EVT, history of venous thromboembolism (VTE), and lower extremity agenesis.

DVT was diagnosed using a published protocol (5) by complete compression duplex ultrasonography of veins in both legs with a high-resolution 7.5 MHz linear-array transducer. The deep veins of the thigh, popliteal fossa, and calf were carefully examined at ~2 cm intervals in the transverse plane. On a more proximal plane, patients were examined in the supine position from the level of the inguinal ligament to the adductor canal. The popliteal vein was examined at its branch in the upper calf. The other calf veins were examined up to the level of the ankle. A diagnosis of deep vein thrombosis was made if duplex ultrasonography examination showed a loss of compressibility of the vein by ultrasound probe pressure, clot, or abnormal flow pattern (loss of phasic flow signal or loss of flow enhancement) with distal compression. The lack of visualization/flow measurements was considered insufficient for interpretation. Ultrasonography was performed in all AIS patients within 10 days of EVT or immediately if the patient complained of throbbing pain, swelling, or warmth in one leg. It should be noted that we included both proximal DVT and distal isolated DVT below the popliteal vein (e.g., posterior and anterior tibial veins, peroneal veins, and the muscular veins) in the study to accurately understand the effects of EVT on DVT in stroke patients.

### EVT procedure and post-operative prevention of DVT

During EVT, 307 and 1 patients underwent puncture at the right and left femoral artery in the groin, respectively. Two patients underwent puncture at the right flexor artery. The catheter was passed to the brain, and the clot was extracted. After EVT, the puncture site in the groin was compressed with a 1 kg sandbag for 6 h, and the punctured lower extremity was immobilized within 24 h of bed rest for 8 h. At 24 h after EVT or at any time within 24 h, neurological deficits progressed, and patients were reexamined with head CT and reassessed with the National Institute of Health Stroke Scale (NIHSS). When there were no hemorrhagic adverse events, regular treatment consisted of antiplatelet drugs that started 24 h after EVT. Three patients received anticoagulation with warfarin or low molecular heparin calcium starting 24 h after EVT because of an existing cardiac valve replacement. Other strategies of treatment included statins, management of blood glucose, blood pressure, or combinations of these treatments, following the Chinese guidelines for the early management of AIS patients (15).

As the bridge therapy with intravenous thrombolysis was considered to improve the recanalization of EVT (16), 68 of 310 AIS patients in our study received a standard intravenous thrombolytic therapy (0.9 mg/kg recombinant tissue-type plasminogen activator, alteplase, over 1 h with 10% of the first bolus) before EVT.

During hospitalization (≤14 days post-EVT), AIS patients were evaluated according to a Chinese guideline (17) and the international guideline (13) and carefully monitored for the risk of both DVT and bleeding. All patients were routinely examined for DVT in the lower extremities by duplex ultrasonography. Our standard protocol for post-thrombectomy management includes a single duplex scan for all stroke patients during their hospitalization. This scan is typically conducted within 2 weeks of EVT, with the timing determined by the individual patient's situation and the examination capacity of our ultrasound department. For example, the examination is scheduled after the patient has been transferred out of the ICU and extubated, taking place in either the stroke unit or the post-stroke unit (normal ward). The examination is only immediately performed if there is a suspicion of DVT. In this study, three patients underwent emergency ultrasound examination.

All the AIS patients who had received EVT had a high risk for both venous thrombosis/embolism and bleeding, so mechanical prophylaxis with intermittent pneumatic compression using the Flowtron® ACS900 Active Compression System (Arjo, Suzhou China) was performed after exclusion of contradictions according to the guidelines (13, 17). The patient wore compression garments (sleeves or boots) on the calves. The compression garment has multiple air chambers that inflate and deflate sequentially, creating an undulating motion that starts from the foot and moves upward, mimicking the natural muscle pump of the legs. Intermittent pneumatic compression (18 h/day) began after EVT, continued throughout the ICU stay, and proceeded to the normal ward if lower extremity muscle strength was less than grade 4 according to the Medical Research Council scale (grade 4: movement against gravity and moderate resistance through almost the entire range of motion) (18).

### Data collection

All clinical information was obtained from the digital history record system. We recorded age, sex, blood pressure, hypertension, diabetes, history of malignant tumors, bridging therapy (intravenous thrombolysis before EVT), EVT operation time (referred to puncture-to-recanalization time), pulmonary infection, respiratory failure, smoking, drinking, coronary heart disease, atrial fibrillation, central venous catheter, mechanical ventilation, time of tracheal intubation, duration of ICU stay, duration of hospital stay, anesthesia mode, endovascular thrombectomy site, lower extremity muscle strength less than grade 3 according to the Medical Research Council Scale (movement against gravity but not against resistance through almost the entire range of motion) (18), NIHSS and modified Rankin Scale (mRS) scores at admission and at discharge, serum creatinine (SCr), glucose, total cholesterol (TC), triglyceride (TG), blood urea nitrogen (BUN), low-density lipoprotein (LDL), high-density lipoprotein (HDL), fibrinogen, and D-dimer. All laboratory tests were conducted within 24 h of the EVT. In addition, we assessed the DVT type.

### Statistical analysis

The statistical analysis of the gathered data was conducted using SPSS software for Windows (Version 26.0, IBM, Armonk, USA). The data for continuous variables were described as mean ± SD, and categorical variables were presented as frequencies. Continuous variables were compared between independent groups by the *T*-test or Mann–Whitney *U*-test depending on whether the continuous variables were normally distributed. Categorical variables were compared by the Pearson χ^2^ test. The independent variables with a *P*-value of < 0.05 in the univariate analysis were included in the multivariable analysis. A multivariable logistic regression study was used to determine the risk factors of DVT and relevant odds ratios (ORs) with 95% confidence intervals. To further investigate the possible influence of female sex on the association between risk factors and the development of DVT, the interaction between operation time, D-dimer, age or lower extremity muscle strength, and female sex was assessed in the logistic regression. To identify the optimal cutoff points for D-dimer plasma level and EVT operation time in predicting DVT, the receiver operator characteristic curve (ROC curve) and the Youden index (J) method were used. The optimal cutoff point was defined with the maximal Youden index (19). A *P*-value of < 0.05 was considered to be statistically significant.

## Results

### Description of DVT characteristics in stroke patients

**Figure 1:**
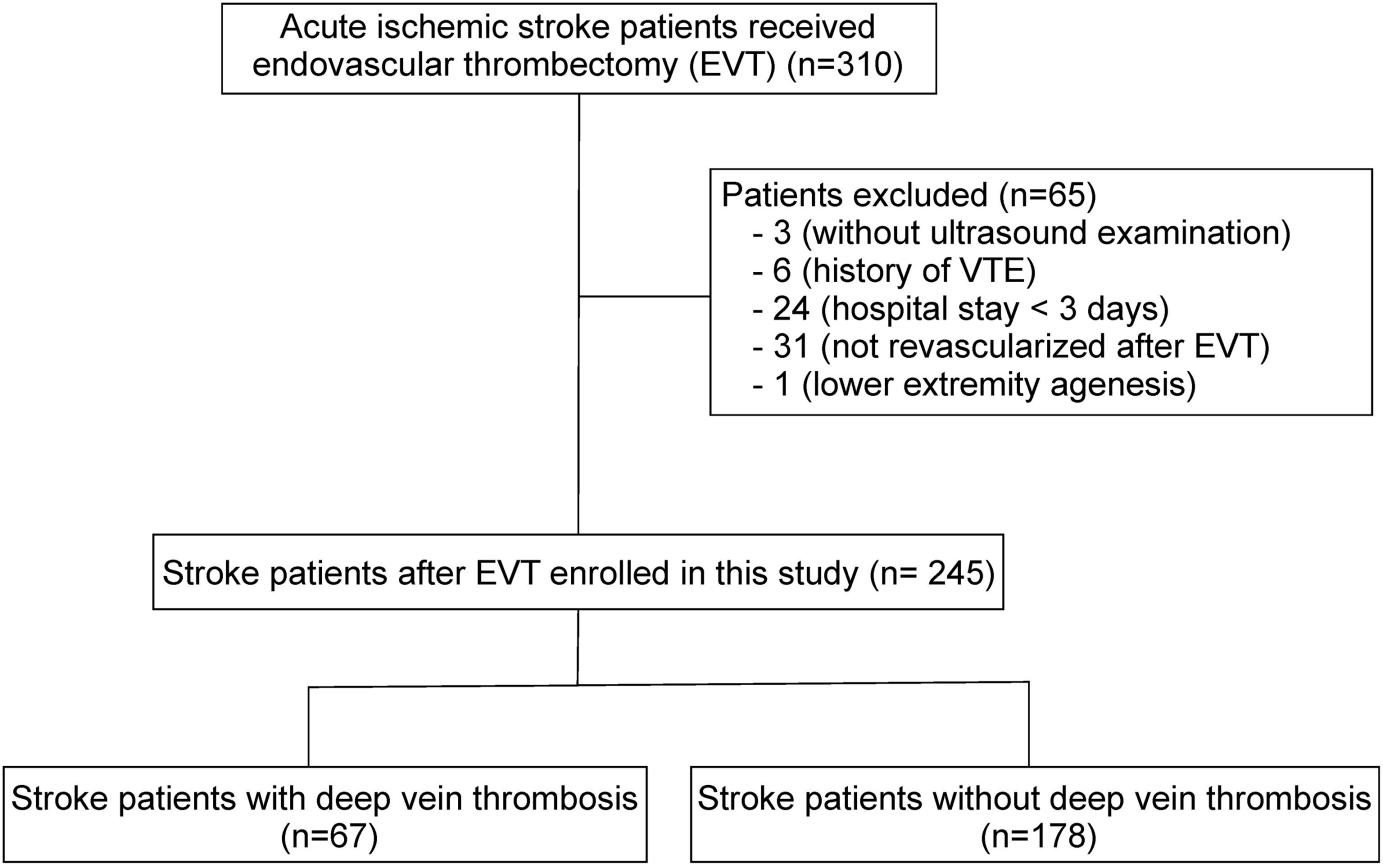
Study flow chart.

From January 2020 to December 2021, 310 AIS patients in our hospital received EVT according to the international and Chinese guidelines (13, 15). Sixty-five patients were excluded due to incomplete information, lack of recanalization at EVT, and other exclusion criteria (Figure 1). Ultimately, 245 patients were enrolled in this study. All patients were administered mechanical prophylaxis using intermittent pneumatic compression immediately after EVT but did not take anticoagulant medications, except that three patients received warfarin or low molecular heparin calcium after EVT because of pre-existing cardiac valve replacement. All patients underwent ultrasonography within 1 to 10 days post-EVT, with a median of 4 days. On the basis of ultrasound findings, DVT was detected in 67 of 245 cases (27.3%), all of whom had undergone right femoral artery puncture during EVT. The prevalence was higher than the overall prevalence previously observed in patients with acute stroke (including ischemic and hemorrhagic stroke) (5–10). Most patients were asymptomatic, and only three of them developed swelling of the lower extremities. No patient developed pulmonary embolism. The most commonly affected blood vessel was the muscular calf vein, which was affected in 57 of the 67 cases (85.07%). This observation was consistent with a published study of asymptomatic DVT in stroke patients (10). The other veins that showed DVT were the posterior tibial vein (5/67), fibular vein (2/67), popliteal vein (1/67), and femoral vein (1/67). In one case (1/67), DVT occurred in multiple vessels.

Of these 67 patients, 29 patients (43.28%) had DVT in the right lower extremity, which was more than 14 patients (20.90%) in the left lower extremity and 24 patients (35.82%) in both lower extremities (Table 1). Of note, 30 (44.77%) patients had lower limb muscle strength less than grade 3 on the left side, 21 (31.34%) on the right side, and 7 (10.45%) on both sides. Nine patients had no lower limb weakness (Table 1). We wondered whether immobilization of the right lower limb during and after surgery contributed to the formation of DVT. We analyzed the correlation between the localization of DVT and the location of lower limb muscle strength less than grade 3. We found that the location of DVT was significantly related to the side of muscle weakness [χ^2^ (6) = 14.783, *P* = 0.022] (Table 1). Thus, the location of the puncture site in the right groin did not seem to be the main influencing factor for DVT.

**Table 1:** Correlation between the locations of muscle weakness and deep vein thrombosis.

	**Location of DVT in lower limbs**			
	**Left**	**Right**	**Both sides**	**Total**
No weakness	2	1	0	3
Weakness on left	9	10	17	36
Weakness on right	2	15	4	21
Weakness on both sides	1	3	3	7
Total	14	29	24	67

Pearson χ^2^ test, P = 0.022; DVT, deep vein thrombosis.

### Univariate predictors of DVT after EVT in AIS patients

To determine the predictors of DVT after EVT, we compared the demographic and baseline characteristics, clinical findings, and therapeutic procedures of patients with and without DVT. Using univariate variable analysis, we found that female, older age, lower extremity muscle weakness <grade 3 (the Medical Research Council scale), respiratory failure, pulmonary infection, central venous catheter, mechanical ventilation, longer duration of EVT surgery, longer duration of ICU monitoring, longer duration of tracheal intubation, and higher plasma D-dimer level positively correlated with an increased risk of developing DVT in AIS patients after EVT (*P*-values for all parameters analyzed <0.05; Table 2). Not surprisingly, the severity of neurological deficits (as shown by NIHSS) was higher in AIS patients who developed DVT on admission than in control patients without DVT, and DVT also negatively affected recovery, as shown by NIHSS and mRS of AIS patients at discharge and 90 days after onset of stroke (*P*-values for all parameters analyzed <0.05; Table 2).

**Table 2:** Characteristics of the patients.

**Variables**	**Total (*n* = 245)**	**Without DVT (*n* = 178)**	**With DVT (*n* = 67)**	***P*-value**
**Demographic characteristics**				
Female, *n* (%)	87 (36)	53 (30)	34 (51)	0.004
Age, mean ± SD, years	67.08 ± 11.67	65.21 ± 12.00	72.06 ± 9.08	<0.001
**Risk factors for stroke**				
Atrial fibrillation, *n* (%)	102 (42)	68 (38)	34 (51)	0.103
Hypertension, *n* (%)	142 (58)	102 (57)	40 (60)	0.846
Diabetes, *n* (%)	42 (17)	27 (15)	15 (22)	0.252
Smoking, *n* (%)	77 (31)	61 (34)	16 (24)	0.159
Drinking, *n* (%)	52 (21)	41 (23)	11 (16)	0.34
Coronary heart disease, *n* (%)	10 (4)	8 (4)	2 (3)	0.732
**Risk factors for DVT**				
Malignant tumors, *n* (%)	15 (6)	11 (6)	4 (6)	0.951
Pulmonary infection, *n* (%)	152 (62)	101 (57)	51 (76)	0.008
Respiratory failure, *n* (%)	27 (11)	13 (7)	14 (21)	0.005
Central venous catheter, *n* (%)	94 (38)	58 (33)	36 (54)	0.004
Mechanical ventilation, *n* (%)	77 (31)	48 (27.0)	29 (43.3)	0.022
Tracheal intubation time, median (P25, P75), days	0 (0, 1.5)	0 (0, 1)	0 (0, 4.25)	0.014
Length of stay in the ICU, median (P25, P75), days	2 (1.5, 4)	2 (1.5, 3)	3 (2, 7)	<0.001
Length of stay in hospital, median (P25, P75), days	11 (8, 14)	10 (8, 13)	14 (10.5, 17.5)	<0.001
Lower limb muscle strength less than grade 3, *n* (%)	177 (72)	113 (63)	64 (96)	<0.001
**Characteristics of endovascular thrombectomy**				
Endovascular thrombectomy site				0.163
Anterior circulation, *n* (%)	194 (79)	137 (77)	57 (85)	
Posterior circulation, *n* (%)	51 (21)	41 (23)	10 (15)	
Mode of anesthesia, *n* (%)				0.48
General anesthesia	74 (30)	51 (29)	23 (34)	
Local anesthesia	171 (70)	127 (71)	44 (66)	
Operation time, median (P25, P75), minutes	60 (45, 95)	55 (44, 85)	85 (59, 110)	<0.001
Bridging therapy, *n* (%)	68 (28)	52 (29)	16 (24)	0.502
**Laboratory findings 24 h after thrombectomy**				
D-dimer, median (P25, P75), mg/L	1.28 (0.7, 2.61)	1.08 (0.6, 1.96)	2.6 (1.38, 4.49)	<0.001
Cr, median (P25, P75), μmoI/L	65 (54, 78)	65 (53.25, 78)	68 (55.5, 78.5)	0.476
BUN, median (P25, P75), mmoI/L	4.97 (3.94, 6.01)	4.87 (3.86, 5.88)	5.03 (4.36, 6.14)	0.167
Glucose, median (P25, P75), mmoI/L	6.21 (5.44, 7.82)	6.18 (5.39, 7.69)	6.37 (5.58, 7.96)	0.232
TG, median (P25, P75), mmoI/L	1.04 (0.77, 1.62)	1.07 (0.77, 1.68)	0.94 (0.73, 1.43)	0.143
TC, median (P25, P75), mmoI/L	3.96 (3.37, 4.61)	3.96 (3.36, 4.68)	3.96 (3.39, 4.47)	0.374
HDL, median (P25, P75), mmoI/L	1.14 (1, 1.33)	1.14 (0.99, 1.3)	1.16 (1.01, 1.38)	0.322
LDL, median (P25, P75), mmoI/L	2.18 (1.71, 2.72)	2.22 (1.74, 2.83)	2.17 (1.58, 2.54)	0.293
Fibrinogen, median (P25, P75), mg/dl	3.14 (2.7, 3.78)	3.14 (2.66, 3.79)	3.17 (2.72, 3.73)	0.696
**Post-operative medication**				0.565
Antiplatelet drugs, *n* (%)	224 (91)	162 (91)	62 (93)	
Anticoagulant drugs, *n* (%)	3 (1.2)	3 (1.7)	0 (0)	
No antiplatelet and anticoagulant drugs, *n* (%)	18 (7.3)	13 (7.3)	5 (7.5)	
**Clinical findings**				
NIHSS at admission, median (P25, P75)	14 (11, 19)	14 (10, 18)	16 (13, 19)	0.025
NIHSS at hospital discharge, median (P25, P75)	7 (2, 13)	5 (2, 11.75)	12 (6.5, 18)	<0.001
mRS score ≤2 at hospital discharge, *n* (%)	69 (28)	60 (34)	9 (13)	0.003
90-day mRS score ≤2, *n* (%)	122 (50)	104 (58)	18 (27)	<0.001

Continuous variables with normal distribution were expressed as mean ± SD, non-normally distributed data were expressed as median (IQR). Cr, serum creatinine; BUN, blood urea nitrogen; TG, triglyceride; TC, total cholesterol; HDL, high-density lipoprotein; LDL, low-density lipoprotein; NIHSS, National Institute of Health stroke scale; mRS, modified Rankin Scale; DVT, deep vein thrombosis.

### Multivariable predictors of DVT after EVT in AIS patients

Because different predictors of DVT in stroke patients identified in the univariate analysis might influence each other, we performed a logistic regression analysis to limit the confounding effects of various variables. The potential predictors, sex, age, lower extremity muscle weakness, respiratory failure, pulmonary infection, central venous catheter, duration of EVT surgery, and plasma level of D-dimer, listed in Table 2, entered the regression analysis. We observed the following variables that independently predicted DVT in AIS patients after EVT: age (OR 1.036, *P* = 0.045), female (OR 3.015, *P* = 0.003), lower limb muscle strength less than grade 3 (OR 7.015, *P* = 0.004), longer EVT time (OR 1.012, *P* = 0.003), and higher D-dimer levels (OR 1.350, *P* < 0.001) (Table 3).

**Table 3:** Multivariable logistic regression analysis of risk factors for DVT after EVT.

**Independent variables**	***P*-value**	**OR**	**95% CI**
Female sex	0.003	3.015	1.446–6.289
Age	0.045	1.036	1.001–1.073
Lower limb muscle strength < grade 3	0.004	7.015	1.887–26.080
Plasma level of D-dimer	0.000	1.350	1.150–1.585
Endovascular thrombectomy time	0.003	1.012	1.004–1.020
Respiratory failure	0.287	-	-
Pulmonary infection	0.654	-	-
Central venous catheter	0.688	-	-

OR, odds ratio; CI, confidence interval.

We wondered whether age, lower limb muscle strength, EVT time, and D-dimer levels differently predicted the development of DVT in women and men. Therefore, we repeated the logistic analysis with these predictors alone, and their combinations with sex (age^*^sex, lower limb muscle strength^*^sex, EVT time^*^sex, and D-dimer level^*^sex) as independent variables. We observed that the interactions between these tested risk factors and sex were not statistically significant (see Supplementary Table 1; *P* > 0.05), which suggested that sex does not alter the associations between age, lower limb muscle strength, EVT time and D-dimer levels, and the development of DVT.

### Determination of cutoff points for EVT time and plasma D-dimer concentrations and their predictive power in the development of DVT

**Figure 2:**
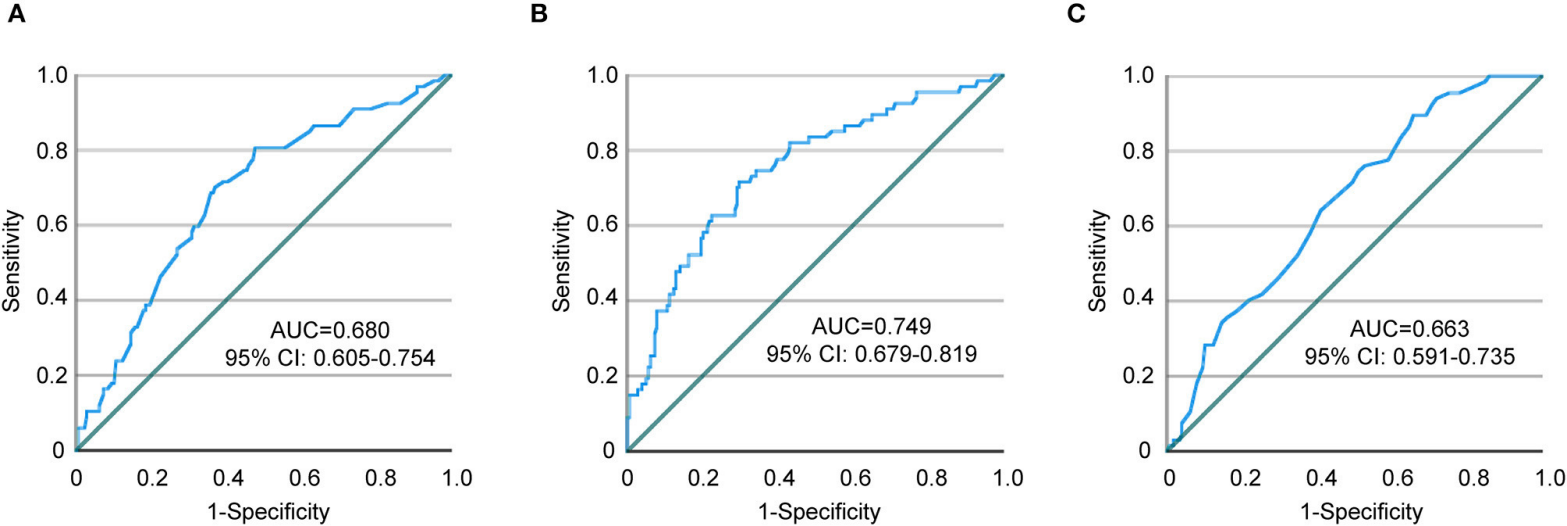
ROC curves showing a relationship between endovascular thrombectomy time, D-dimer, age, and DVT. **(A)** Endovascular thrombectomy time, **(B)** D-dimer, and **(C)** Age. ROC, receiver operating characteristic; AUC, area under the curve; CI, confidence interval.

As described above, our study revealed that aging, female sex, lower limb paralysis, and higher plasma D-dimer levels were associated with the development of DVT, which is consistent with previous research findings (5–8). In the specific cohort of stroke patients with occlusion in a large cerebral artery and receiving thrombectomy, we identified the operative time of EVT as another independent risk factor for DVT. As EVT potentially alters the coagulation and thrombolytic activity in stroke patients (14, 20, 21), the threshold of D-dimer level in predicting/diagnosing DVT should be updated. In the following study, we used the ROC curve and defined the cutoff point for the operative time of EVT as 65.5 min with a sensitivity and specificity of 70.1 and 62.9%, respectively (Table 4; Figure 2A). Similarly, the cutoff point for D-dimer concentration was 1.62 mg/L with a sensitivity of 71.6% and specificity of 70.2% (Table 4; Figure 2B), which is consistent with published results with the optimal cutoff points at 1.52, 1.59, and 1.66 mg/L for predicting DVT in acute stroke patients (9, 22, 23). We also defined the cutoff point for age as 60.5 years with a sensitivity and specificity of 89.6 and 34.3%, respectively (Table 4; Figure 2C). Due to the low specificity, we calculated age as a continuous variable in the subsequent statistical analysis.

**Table 4:** Determination of cutoff points for EVT time and D-dimer with ROC curve.

**Variables**	**Cutoff point**	**Sensitivity**	**Specificity**	**Youden index**	**AUC**	**95% CI**	***P*-value**
Endovascular thrombectomy time	65.5	0.701	0.629	0.330	0.680	0.605–0.754	<0.001
D-dimer	1.62	0.716	0.702	0.418	0.749	0.679–0.819	<0.001
Age	60.5	0.896	0.343	0.239	0.663	0.591–0.735	<0.001

AUC, area under curve; CI, confidence interval.

We then repeated the logistical regression to assess the predicting power of prolonged operative time during EVT (≥65.5 min) and higher plasma D-dimer level (≥1.62 mg/L) for the development of DVT. Interestingly, prolonged operative time and higher plasma D-dimer levels were consistently the independent risk factors for the development of DVT in stroke patients after thrombectomy (Table 5). The odd ratios (>4) were even larger than those calculated from the continuous variables EVT time and D-dimers (see Table 3). Age was excluded from the list of risk factors in this analysis (*P* > 0.05). Respiratory failure, pulmonary infection, and central venous catheter were not independent predictors for the development of DVT (*P* > 0.05).

**Table 5:** Multivariable logistic regression analysis to determine the predictive power of long EVT time and high D-dimer level for DVT.

**Independent variables**	***P*-value**	**OR**	**95% CI**
Female sex	<0.001	3.569	1.729–7.367
Age	0.134	-	-
Lower limb muscle strength less than grade 3	<0.001	10.732	2.967–38.818
Longer endovascular thrombectomy time (≥65.5 min)	<0.001	4.388	2.166–8.889
Higher D-dimer (≥1.62 mg/L)	<0.001	5.055	2.495–10.243
Respiratory failure	0.383	-	-
Pulmonary infection	0.676	-	-
Central venous catheter	0.787	-	-

OR, odds ratio; CI, confidence interval.

## Discussion

DVT has been studied as a common complication in stroke patients over the past 50 years (24). In the era of thrombectomy, we are now addressing this old but new question. AIS patients after endovascular thrombectomy were at potentially high risk for DVT. Prolonged surgical time for thrombectomy, along with other known risk factors, such as age, female sex, lower extremity muscle weakness, and higher plasma D-dimer levels, was strongly associated with the development of DVT.

Our study showed that the prevalence of DVT in ischemic stroke patients within 10 days of thrombectomy was 27.3% (67/245), which is higher than the overall prevalence of 11.5 to 22.1% in stroke patients (including ischemic and hemorrhagic stroke, with and without intravenous thrombolysis) within 14 days of disease onset (5–10). Our prevalence included both proximal and distal DVT, and 85% of patients had DVT in the muscular calf veins. Compared with proximal DVT, distal DVT is less likely to cause severe clinical sequelae, i.e., pulmonary embolism (25). However, it does not mean that distal DVT is clinically insignificant. It is still possible for thrombosis in the muscular calf veins to progress further into the deep venous trunk and lead to more severe DVT (26, 27).

Disease severity may be a reason for the higher prevalence of DVT in our study. Our patients had higher NIHSS scores (with a median of 14) compared with other populations studied (5–10). It is widely recognized that paralysis in stroke patients promotes the development of DVT (24). We also observed that lower extremity muscle weakness was the strongest independent predictor of DVT (see Tables 3, 5). The loss of venous pump function caused by muscle contusion may contribute to DVT formation. In clinical practice, we encourage patients to actively move both their paralyzed and non-paralyzed extremities and to get out of bed whenever possible. Additionally, we advise caregivers to passively move the patient's paralyzed extremities.

Given the potential cause–effect relationship between paralysis and DVT, we were curious about whether the immobilization of lower limbs during and after EVT surgery could lead to the development of DVT. Surprisingly, a transfemoral puncture in the right groin did not alter the correlation between the localization of DVT and the side of muscle weakness. This suggests that immobilization of the right lower extremity during and after EVT surgery does not contribute significantly to the formation of DVT. Nevertheless, it is reasonable to choose an optimal vascular access for EVT. For endovascular interventions in patients with acute coronary syndromes or ischemic stroke, the transradial approach has been shown to be a safe alternative to the transfemoral approach (28–30).

As far as we know, we are the first to report that the time from puncture to reperfusion in the EVT procedure is an independent risk factor for the development of DVT in stroke patients, with a longer duration associated with a higher risk. A longer time from puncture to reperfusion in stroke patients has been already associated with poor recovery within 3 months and an increase in malignant cerebral edema and symptomatic intracranial hemorrhage within 24 h after successful EVT (31–33). Our study indicated that prolonged performance of EVT not only affects the brain locally but also systemically affects the coagulation system. The facilitated development of DVT may subsequently hinder the rehabilitation of paralyzed legs and lead to poor recovery of stroke patients described above (31). However, it is unclear whether and how EVT itself promotes thrombosis in stroke patients. Experiments in artificial blood vessels and animal models showed that EVT has the potential to damage endothelial cells and vessel walls, which is exacerbated by repeated stentriever passages (14, 20). The damaged endothelial cells and blood walls release tissue factors and subsequently activate the coagulation cascade (34). Indeed, a small study showed that the procoagulant activity of tissue factor and plasminogen activator inhibitor-1 increased after EVT in patients with ischemic stroke (21).

D-dimer is a small protein fragment present in the blood after a blood clot has been degraded by fibrinolysis. D-dimer is an important diagnostic marker for venous thromboembolism (35). We observed that plasma D-dimer levels increased within 24 h after EVT and independently predicted DVT. The plasma level of D-dimer has been extensively investigated as a predictor for DVT in stroke patients. Interestingly, the cutoff point for D-dimer in predicting DVT in our study (1.62 mg/L) is very similar to the cutoff points (1.52, 1.59, and 1.66 mg/L) found in three other studies that measured D-dimer levels in acute stroke patients upon admission (9, 22, 23). This suggests that a high level of D-dimer in plasma may be a reliable biological marker for diagnosing DVT. It is worth noting that the subjects in these three studies did not undergo EVT, indicating that thrombectomy may not significantly affect the plasma level of D-dimer.

The prevention of venous thromboembolism is challenging. To date, there is no standard clinical practice guideline. The clinical trial of peri-EVT treatment with aspirin and/or unfractionated heparin was discontinued because these measures significantly increased the risk of intracranial hemorrhage (36). We have used intermittent pneumatic compression to prevent DVT in most stroke patients after EVT because this method has been shown to reduce the risk of DVT and potentially improve survival in a large number of patients who are immobile after stroke (37). Indeed, the prevalence of proximal DVT in the stroke patients we studied was very low [of 245 stroke patients after EVT, only one patient developed DVT in the popliteal vein (1/245; 0.41%) and one in the femoral vein (1/245; 0.41%)], compared with published prevalence, e.g., 8.30% (6) and 2.43% (10), which could significantly reduce the life-threatening risk of DVT. However, another study including stroke patients with and without intermittent pneumatic compression is needed to evaluate the therapeutic efficacy of mechanical prophylaxis in preventing DVT after EVT.

Our study has several limitations. First, because of emergent mechanical thrombectomy, ultrasound was not performed prior to EVT to determine whether the patient had already developed DVT at the time of admission. Nevertheless, the observation that the prevalence of DVT was related to the time of thrombectomy is plausible because the prolonged time of thrombectomy was unlikely due to the preexistence of DVT. Our finding is scientifically important because it demonstrated that thrombectomy may induce a thrombotic event although the causal relationship and its clinical significance require further investigation. Second, the study population was chosen from a single institution with a limited number and diversity of patients. Third, it was a retrospective study, and some data may not have been collected. In addition, we did not perform further validation to determine whether people with all these risk factors are more likely to develop DVT.

## Conclusion

Our study shows that ischemic stroke patients who receive endovascular mechanical thrombectomy may be at increased risk of developing deep vein thrombosis, especially if they are old (≥60.5 years), female, have weak lower extremity muscles (<grade 3 in the Medical Research Council scale), have had the thrombectomy for a long time (≥65.5 min), and have a high plasma level of D-dimer (≥1.62 mg/L). Prophylaxis for deep vein thrombosis should be given as soon as possible after thrombectomy. However, the results of our study need to be validated by a multicenter prospective study with a larger population of stroke patients.

## Data availability statement

The original contributions presented in the study are included in the article and Supplementary material, further inquiries can be directed to the corresponding authors.

## Ethics statement

The studies involving humans were approved by Ethics Committee of Taizhou Hospital, Zhejiang Province, China. The studies were conducted in accordance with the local legislation and institutional requirements. Written informed consent for participation was not required from the participants or the participants' legal guardians/next of kin because the project was a retrospective study, collecting data from a digital history recording system.

## Author contributions

LH, FW, and YL conceived and designed the study. W-YQ and YL designed the statistical analysis. LH, T-HT, and J-MY collected and analyzed the data and prepared the figure and tables. LH, FW, YL, J-MY, W-YQ, and XP-X wrote and revised the manuscript. All authors approved the final version of the manuscript.
