# Supplementary material for: Risk factors for lower extremity deep vein thrombosis in acute stroke patients following endovascular thrombectomy: a retrospective cohort study
Source: Front Neurol. 2023 Oct 11;14:1249365. doi: 10.3389/fneur.2023.1249365 (PMC10599242; doi:10.3389/fneur.2023.1249365)
Supplement: Supplementary file 1 [file Data_Sheet_1.PDF]

Supplementary Table 1, Multivariable logistic regression analysis of risk factors and their interactions with female sex

	Regression coefficient B	Standard error	Wald	df	*P*-value	OR	95% C.I. for OR	
							Lower	Upper
Female sex	0.289	3.006	0.009	1	0.923	1.335	0.004	483.477
Age	0.032	0.022	2.164	1	0.141	1.032	0.989	1.077
EVT time	0.012	0.005	6.927	1	0.008	1.012	1.003	1.021
Muscle weakness < grade 3	2.391	1.084	4.870	1	0.027	10.926	1.307	91.360
D-dimer	0.275	0.097	8.010	1	0.005	1.316	1.088	1.592
Sex by age	0.010	0.038	0.069	1	0.792	1.010	0.938	1.087
EVT time by sex	0.002	0.010	0.039	1	0.844	1.002	0.983	1.022
Muscle weakness by sex	-0.444	1.362	0.106	1	0.744	0.641	0.044	9.257
D-dimer by sex	0.136	0.200	0.465	1	0.495	1.146	0.775	1.695
Constant	-7.242	1.886	14.747	1	0.000	0.001		

Note: OR, Odds ratio; CI, confidence interval
